# Graphene oxide/mussel foot protein composites for high-strength and ultra-tough thin films

**DOI:** 10.1038/s41598-020-76004-6

**Published:** 2020-11-05

**Authors:** Eugene Kim, Xuyan Qin, James B. Qiao, Qingqing Zeng, John D. Fortner, Fuzhong Zhang

**Affiliations:** 1 Department of Energy, Environmental and Chemical Engineering, Washington University in St. Louis, One Brookings Drive, Saint Louis, MO 63130 USA; 2 Department of Chemical and Environmental Engineering, Yale University, New Haven, CT 06520 USA; 3 Institute of Materials Science and Engineering, Washington University in St. Louis, Saint Louis, MO 63130 USA; 4 Division of Biological and Biomedical Sciences, Washington University in St. Louis, Saint Louis, MO 63130 USA

**Keywords:** Molecular biology, Engineering, Materials science

## Abstract

Graphene oxide (GO)-based composite materials have become widely popular in many applications due to the attractive properties of GO, such as high strength and high electrical conductivity at the nanoscale. Most current GO composites use organic polymer as the matrix material and thus, their synthesis suffers from the use of organic solvents or surfactants, which raise environmental and energy-consumption concerns. Inspired by mussel foot proteins (Mfp) secreted by the saltwater mussel, *Mytilus galloprovincialis* and by recent advances in microbial protein production, we developed an aqueous-based green synthesis strategy for preparing GO/Mfp film composites. These GO/Mfp films display high tensile strength (134–158 MPa), stretchability (~ 26% elongation), and high toughness (20–24 MJ/m^3^), beyond the capabilities of many existing GO composites. Renewable production of Mfp proteins and the facile fabrication process described provides a new avenue for composite material synthesis, while the unique combination of mechanical properties of GO/Mfp films will be attractive for a range of applications.

## Introduction

Graphene has become a widely studied material that has the potential to be used in a wide variety of applications, including electronics, photovoltaics, semiconductors, water treatment, and multifunctional textiles, among others^1–5^. The unique two-dimensional atomic arrangement of carbon in graphene gives rise to many of its attractive properties, such as electrical and thermal conductivities, flexibility, and high-strength^6,7^. As a graphene derivative, graphene oxide (GO) shares many attractive properties with graphene and can be more easily synthesized. Furthermore, owing to an abundance in oxygen-containing groups on both its basal and edge planes, GO is more soluble in polar solvents and can be readily functionalized, underpinning broad applicability, particularly in nanocomposites with enhanced mechanical, electrical, and physicochemical properties^8–11^. GO has been shown to be an exceptional building block for the fabrication of new composite materials with enhanced mechanical properties. Chemical crosslinking with polymer matrices has been one commonly utilized method for achieving this goal^12–14^. Most GO-based composites use organic polymer as the matrix material^15–18^. However, due to material incompatibility between GO with most organic polymers, it is difficult to obtain a homogenous single phase mixture when preparing the composites^19^. As a result, a large amount of organic solvents or surfactants are often needed in industrial-scale processes, which raises concerns in scalability, process safety, toxicity, and energy usage.

More recently, biological materials, such as proteins and protein-like materials, have been used as matrix materials in GO-based composites, due to their amphiphilic nature and ability to withstand high mechanical forces^20–23^. Proteins can either be isolated directly from natural resources or recombinantly produced from renewable feedstock, and they can be degraded, thus offering a sustainable route for both material synthesis and end-of-life management. Unlike organic polymers, proteins are often monodisperse, have controllable sequences and structures, and have a wider range of chemistries. Previously, soy protein isolate and silk fibroin have both been used to form GO composites^24,25^. These proteins contain secondary structures, such as α- and β- helices in corn zein or β-sheets in silk fibroin. The hydrophobic effect drives the formation of these secondary structures; however, when interacting with GO nanosheets, proteins tend to change conformation to redistribute amino acid residues, adopting a new set of entropically favored interactions, such as hydrogen bonding and electrostatic interactions. In some cases, such proteins undergo denaturation and aggregation in the presence of GO, which would lead to undesirable mechanical properties^26^.

One unique class of proteins that has not been fully explored with regard to composite synthesis is the intrinsically disordered family of mussel foot proteins (Mfp). Naturally secreted by the marine mussel, Mfp utilize a wide range of molecular interactions to bond to hydrophilic surfaces such as rocks, metals, and glass, as well as hydrophobic surfaces, such as plastics. These strong interactions with surfaces are achieved largely in part due to the side chain of the non-canonical amino acid, 3,4-dihydroxyphenylalanine (DOPA). More interestingly, the tight interaction between Mfp and various surfaces take place underwater. If this aqueous-based molecular bonding can be used to prepare composite materials, it will provide a low-energy, environmentally-friendly process for composite fabrication, which would otherwise involve high temperature processes or organic solvents compatible with organic polymers.

Herein, we describe a facile approach for fabricating GO composite films using Mfp through an aqueous-based processing route. The Mfp were recombinantly synthesized by genetically engineered *E. coli* with a subsequent post-translational modification step for the generation of DOPA residues^27^. We further developed an aqueous procedure to fabricate free-standing GO/Mfp films. These composite films consisted of up to 20 w/w% of Mfp and displayed high strength and toughness, comparable to or even stronger than previously reported GO composites.

## Results

### Synthesis of graphene oxide/mussel foot protein composite films

**Figure 1:**
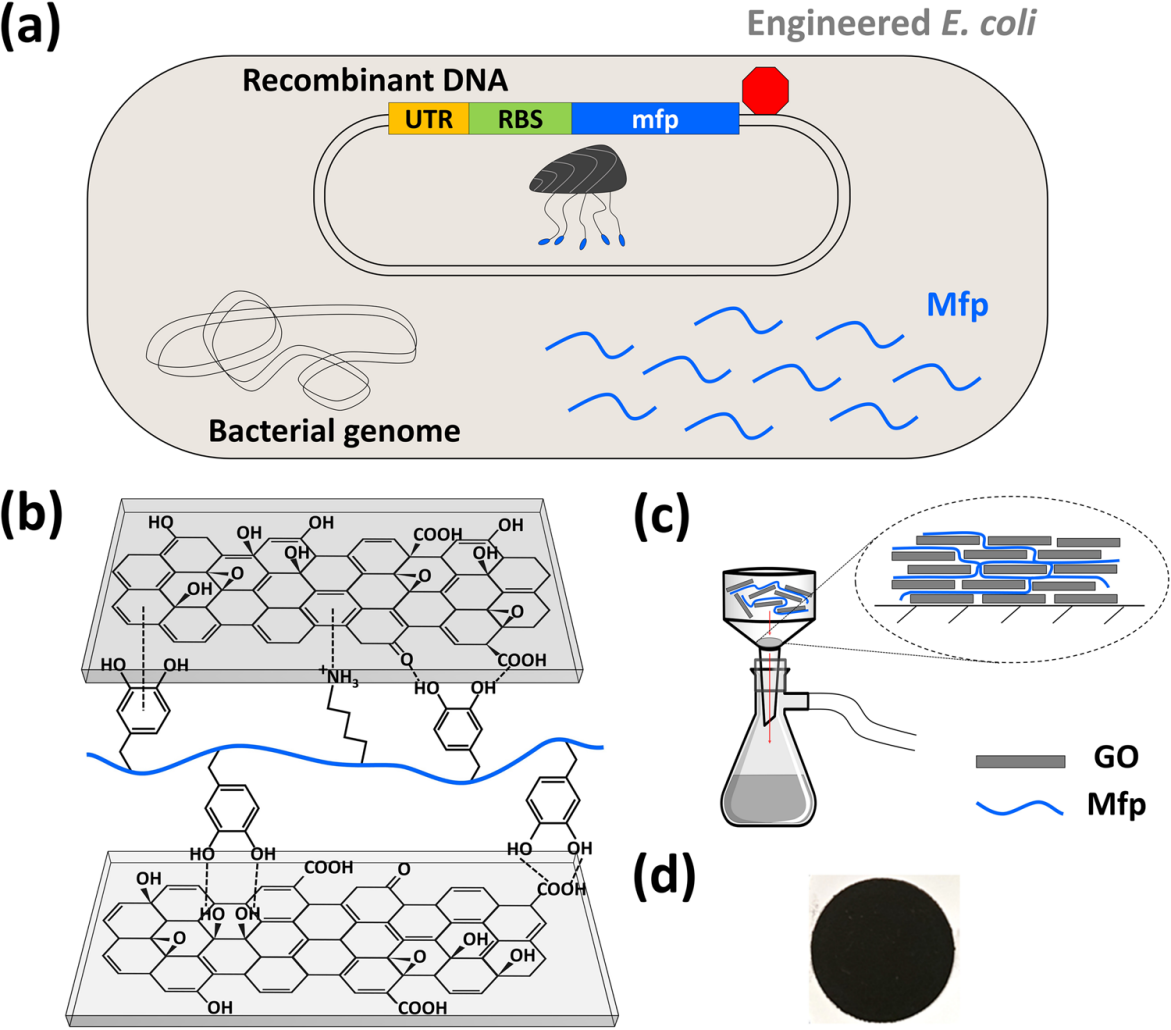
Synthesis of graphene oxide/mussel foot protein composite. (**a**) *E. coli* were genetically engineered to overexpress mussel foot protein (Mfp) in vivo. Proteins were extracted, purified downstream, and underwent post-translational steps, such as splicing into larger molecular weights and reacting with tyrosinase. (**b**) Schematic showing DOPA and lysine residues on recombinant Mfp interacting with oxygen-functional groups on graphene oxide (GO) nanosheets. (**c**) Schematic showing the experimental set-up involving vacuum filtration of a homogenous GO/Mfp mixture onto a PES support membrane, resulting in a thin film composite. (**d**) Photograph of resulting GO/Mfp composite material.

We hypothesize that the unique chemistry of Mfp allows the flexible protein chains to form extensive interactions with GO nanosheets through hydrophobic interactions, π–π stacking (via Mfp aromatic side chains), cation–π interactions, and hydrogen bonding via DOPA-alcohol, DOPA-carboxylate, and bi-DOPA pairs (Fig. 1a,b)^28–31^. To promote a robust network of interactions between GO and Mfp, we chose Mfp5 from *Mytilus galloprovincialis* due to its high DOPA content and overall positive charge, which can reduce Mfp–Mfp interactions in low ionic strength solvents while promoting electrostatic interactions with the negatively-charged GO nanosheets^32^. Mfp can accomplish the formation of an interaction network in water-rich solvents, which eliminates the need to use harsh organic solvents. Although some Mfp3 peptides have been shown to form coacervates in aqueous solutions^33^, Mfp5 precipitates at neutral pH and basic conditions, probably due to its higher molecular weight^27^. However, at acidic pH levels, our Mfp5 remains soluble. We thus used pH 4.5 acetate buffer to prepare the Mfp5/GO mixtures. The acidic condition also helps prevent the catechol groups from oxidizing. Additionally, we hypothesized that a longer Mfp chain length will participate in extensive molecular interactions with GO, therefore strengthening the composite’s molecular network and resulting in better film mechanical properties^27,34,35^. Thus, Mfp5^(3)^, a synthetic protein containing three consecutive repeats of Mfp5 was also used in this study^27,36^.

Both Mfp5 and Mfp5^(3)^ were microbially synthesized by genetically engineered *E. coli*, purified, and enzymatically modified to convert tyrosine residues to DOPA using our previously developed method^27^. To fabricate the GO/Mfp composites, different ratios of GO and Mfp were mixed to identify the condition, in which both components stayed soluble in aqueous solution. We found that at a GO-to-Mfp ratio of 5:1 and a pH of 4.5, the solution was homogenous after sonication. The solution was then vacuum filtered and dried to obtain free-standing thin films (Fig. 1c). To evaluate whether longer periods of stacking and compacting could lead to mechanically stronger films, different filtration times were used to compare composite films. All of our composite films were black in color and had metallic lusters (Fig. 1d), similar to GO/polymer composites examined in previous studies^37,38^.

### Structural characterization of composite films

**Figure 2:**
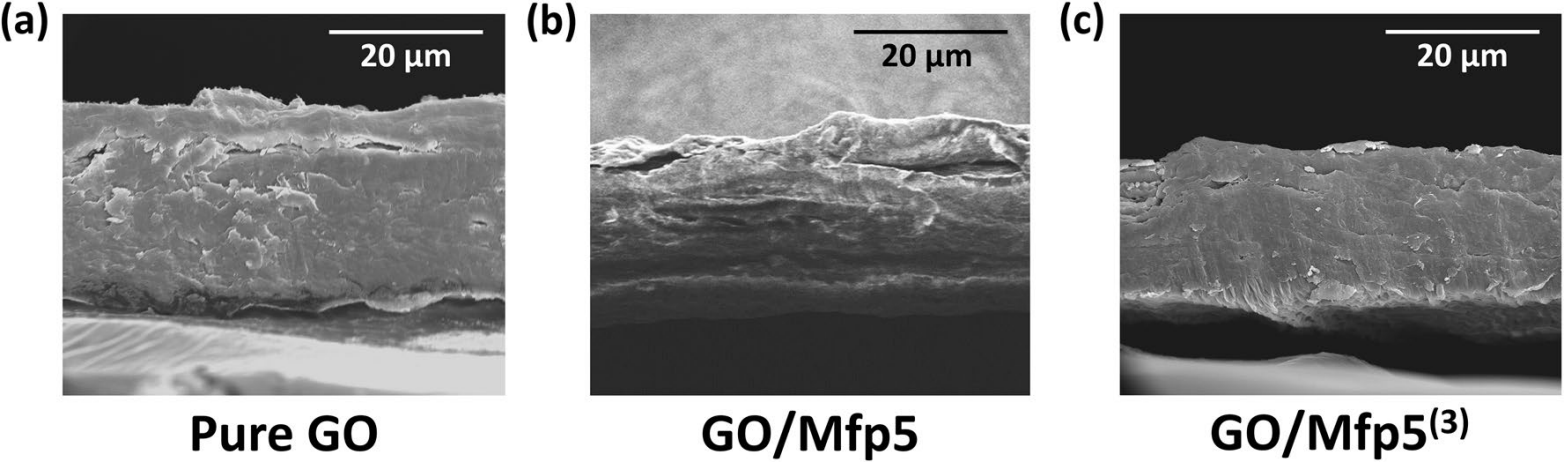
SEM images of cross sections of different films. (**a**) Pure GO film. (**b**) GO/Mfp5 film. (**c**) GO/Mfp5^(3)^ film. There is approximately 5 mg of GO in each film.

Micro-scale structures of the composite films were first studied using scanning electron microscopy (SEM). The cross-sections of the GO, GO/Mfp5 and GO/Mfp5^(3)^ films all show dense packing with minimal voids and cracks, which would otherwise negatively affect mechanical strength via crack propagation (Fig. 2). Both GO/Mfp5 and GO/Mfp5^(3)^ films have comparable thicknesses of 25.1 ± 2.6 and 24.8 ± 4.9 μm, respectively; however, these films were both slightly thinner than pure GO films (27.8 ± 2.9 μm, *P* < 0.05). In addition, we found that filtration time did not affect overall film thicknesses (Supplementary Figure S1).

**Figure 3:**
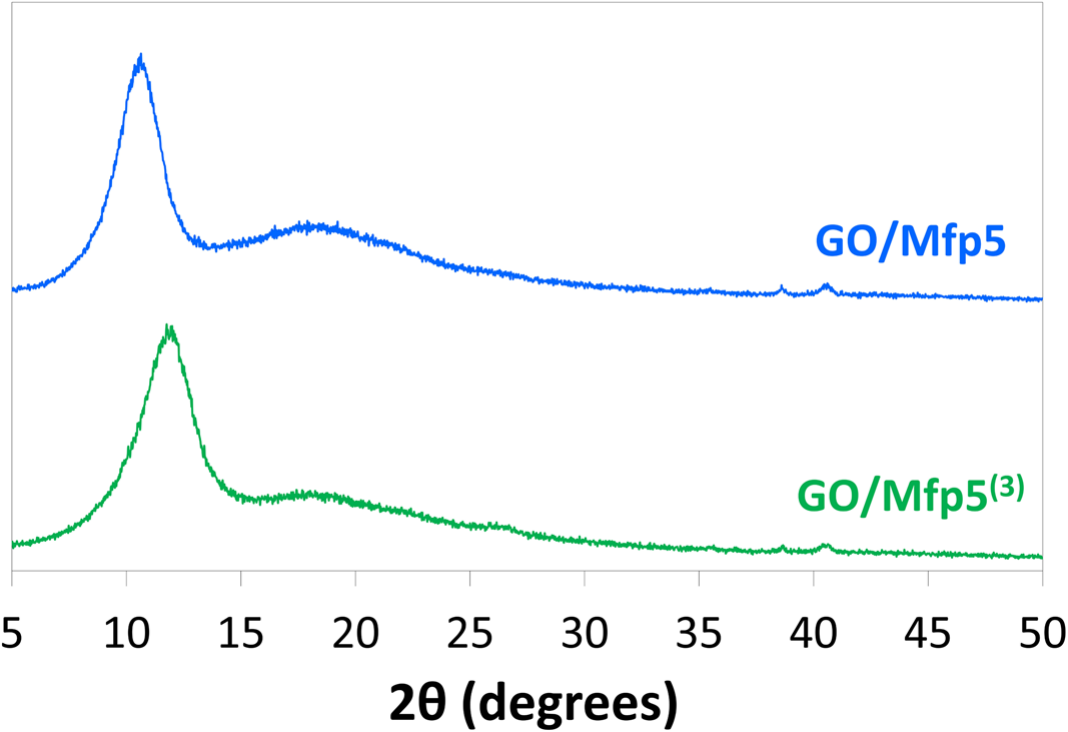
X-ray diffraction pattern of GO/Mfp film composites. The addition of Mfp and an increase in Mfp molecular weight shifts the characteristic 2θ peak of GO, which shows a decrease in interplanar distance between GO nanosheets.

X-ray diffraction (XRD) analysis was conducted to examine the extent of nanosheet alignment within the films and interlayer spacing between nanosheets. Previous studies of pure GO films showed characteristic peaks at 2θ values of 10–11^12,39^. Similar peaks at 10.6° and 11.8° were observed in our GO/Mfp5 and GO/Mfp5^(3)^ films, respectively (Fig. 3), suggesting good alignment of GO nanosheets in the composite films. The average interplanar distances, d, between GO nanosheets were estimated using the measured 2θ values. The d values of GO/Mfp5 and GO/Mfp5^(3)^ were 8.1 Å and 7.4 Å, respectively, and were slightly lower than the 8–9 Å interplanar distance of pure GO measured here and in previous studies^40–42^. Decreased interplanar distance is likely to be caused by the Mfp’s natural ability to displace water molecules hydrating intercalated space between GO nanosheets, a layer that has been shown to be as thick as 1.2 nm^32,43–46^. This is consistent with the decreasing trend in film thicknesses as observed in SEM and consistent with the decreased interplanar distance as molecular weight of Mfp increases.

Thermogravimetric analysis (TGA) was further conducted to confirm Mfp incorporation in our composite films, as well as to test their thermal stabilities. Our pure GO film exhibited similar thermal degradation kinetics as other studies, for which there is a rapid decrease in mass at around 200 °C due to evaporation of associated water molecules (Supplementary Figure S2)^9,47^. Pure lyophilized proteins, which were used as single-component controls, degraded rapidly at temperatures higher than ~ 150 °C (Supplementary Figure S3). However, the proteins that were incorporated into our GO films do not contribute to a higher rate of degradation in the overall film and remain stable within the range of 200–550 °C, which is also beneficial for many practical applications. Fourier transform infrared (FTIR) spectra of our pure GO and GO/Mfp films closely resemble that of pure graphene oxide (Supplementary Figure S4). Additionally, the FTIR spectra show that there were no peak shifts or significant changes in peak intensities throughout the measured wavenumber range, suggesting the lack of covalent interactions between GO and Mfp as well as a lack of new protein secondary structures induced during composite formation. Rather, the Mfp molecules bind on and between the GO nanosheets through non-covalent interactions.

### Mechanical properties of composite films

**Figure 4:**
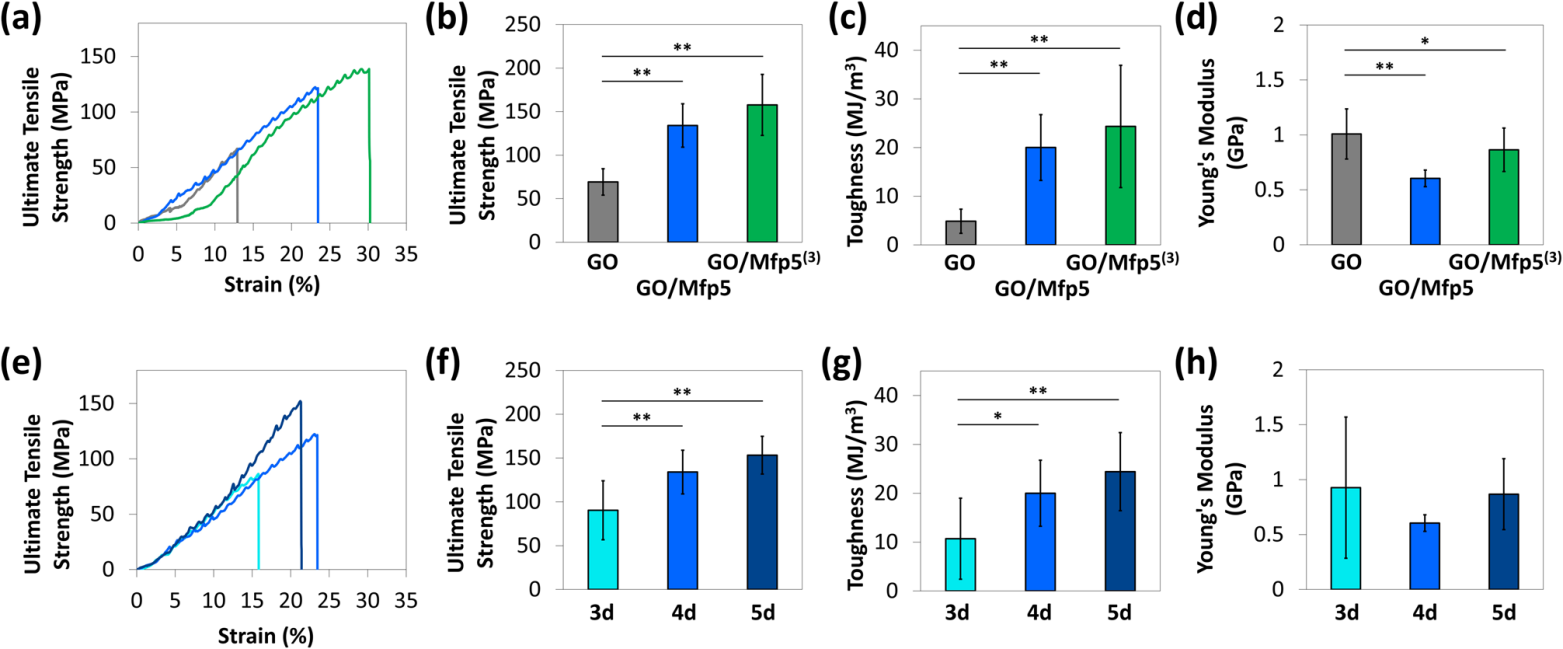
Mechanical properties of pure GO and GO/Mfp films. (**a**) Representative stress–strain curves for pure GO (gray), GO/Mfp5 (blue), and GO/Mfp5^(3)^ (green) films filtered for 4 days. (**b**) Ultimate tensile strength, (**c**) Toughness, and (**d**) Young’s modulus were calculated and plotted for these three film types. (**e**) Representative stress–strain curves for GO/Mfp5 films filtered for 3 days (light blue), 4 days (blue), and 5 days (dark blue). (**f**) Ultimate tensile strength, (**g**) Toughness, and (**h**) Young’s modulus were calculated and plotted for the GO/Mfp5 films that were filtered for different lengths of time. Statistically significant differences (one-way ANOVA with Tukey’s HSD post hoc analysis) are indicated by a single asterisk (*P* < 0.05) or double asterisks (*P* < 0.01).

Standard tensile testing was performed on rectangular-shaped strips of as-synthesized films. We obtained the ultimate tensile strength, toughness, and Young’s modulus of each film from the measured stress–strain curves (Fig. 4a,e). Compared to pure GO film, GO/Mfp5 composite film displayed a 1.9- and 4.1-fold higher tensile strength and toughness, respectively, and 40% decrease in Young’s modulus. Thus, Mfp is participating in the formation of an extensive interaction network, allowing the films to withstand higher stress before fracture and to absorb a higher amount of energy before deformation. We also observed that incorporating the higher molecular weight Mfp5^(3)^ further enhanced ultimate tensile strength by 2.3-fold and decreased Young’s modulus by 14% with respect to the pure GO film control, while maintaining toughness at a similar level to GO/Mfp5 films (Fig. 4b–d). Consistent with our design, the higher tensile strength of the GO/Mfp5^(3)^ compared to the GO/Mfp5 film indicates the formation of a stronger protein-GO interaction network. Further, the observed higher strain of the GO/Mfp composite compared to that of GO film suggests that under tensile stress, the Mfp protein chains are straightened, sliding along the GO nanosheets, thereby absorbing energy and contributing to a higher film toughness. Additionally, we fabricated GO/Mfp5 films with varied filtration times. We hypothesized that longer filtration times could potentially increase nanosheet alignment and promote tighter packing of the nanosheets. Indeed, films filtered for 4 days exhibited a 1.5-fold higher tensile strength and 1.9-fold higher toughness, than those of films filtered for 3 days. When films were filtered for 5 days, no further enhancement on film strength and toughness was observed, suggesting an optimal filtration time was reached (Fig. 4f–h).

**Figure 5:**
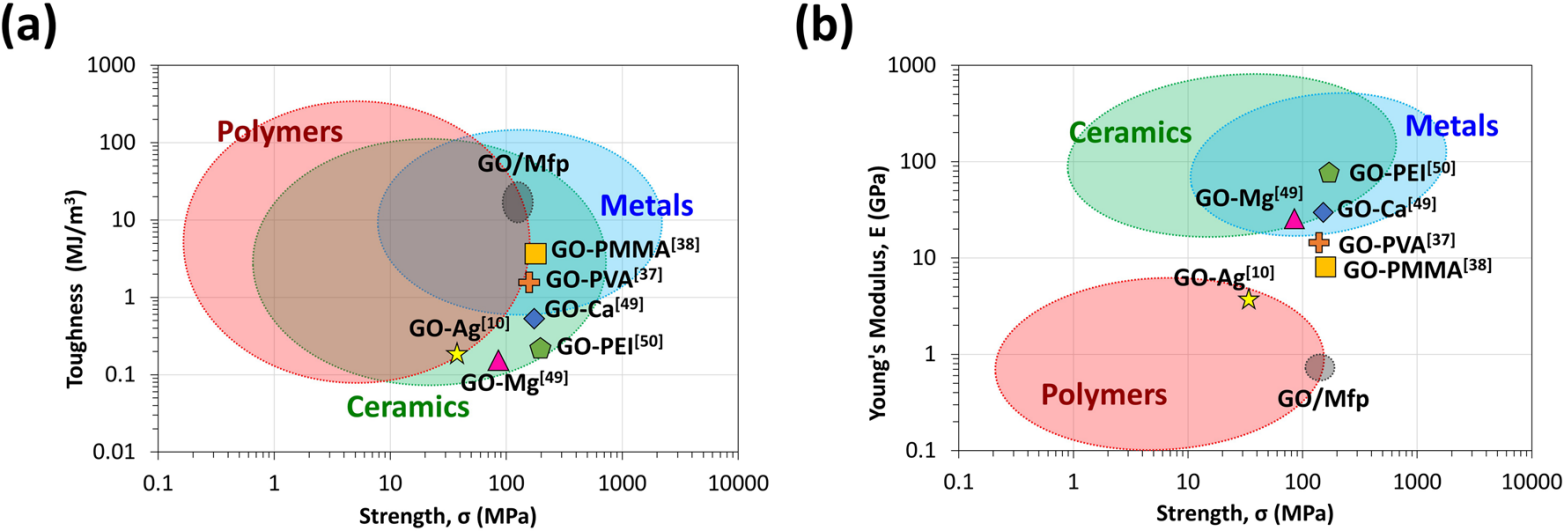
Ashby plots showing the strength, Young’s modulus, and toughness of different classes of materials and GO composite materials. (**a**) GO/Mfp films synthesized in this study are compared to the strength and toughness of other materials, such as polymers, metals, and ceramics, as well as other graphene oxide composites. (**b**) GO/Mfp films synthesized in this study are compared to the strength and Young’s modulus of other classes of materials and composites^10,37,38,48–50^.

Our approach for synthesizing our GO composites allowed us to create films that were significantly tougher than polymeric materials while comparable in tensile strength to many types of metals, metal alloys, and ceramics, as well as other GO composites synthesized with divalent ions or synthetic polymer crosslinkers (Fig. 5). The toughness of our films is 1–2 orders of magnitude higher than those of similar GO composites. The unique Mfp matrix endowed the GO/Mfp films with the ability to absorb a large amount of energy to deform without fracturing. This high material toughness may open new applications in protection and energy absorption (Fig. 5a). It is also important to note that that the GO/Mfp films also have lower Young’s moduli, which make them less stiff and more flexible due to the use of soft protein matrices (Fig. 5b)^10,37,38,48–50^. Such a combination of toughness and flexibility is highly desired in environmental GO applications, for which there are many^51–53^. When subject to reducing conditions, our film was moderately conductive, exhibiting conductivities of 0.6–1.5 S/m. If further processed and optimized, our film, given its flexible nature could potentially be used in bioelectronic applications, for example, as a wearable device that can convert and transmit physical resistance into electrical signals^54^. Such property combinations underpin GO/Mfp films as a unique material, demonstrating properties that are not possible through existing GO composite strategies.

## Conclusions

In summary, we report a new type of GO composite material using Mfp as a novel matrix. Mfp matrices were microbially synthesized from renewable feedstock, and the composite film was made through an environmentally-friendly, aqueous-based process route. As shown, GO/Mfp composites integrate the unique chemistry of Mfp, as well as the versatility of GO. These GO/Mfp films have low stiffness, high tensile strength, and ultra-high toughness, comparable to or exceeding previously reported GO-based materials^55^. The simple green synthesis process will also open new avenues for composite preparation, and when coupled with unique mechanical properties of the GO/Mfp, material adoption is thus attractive for a variety of applications.

## Methods

### Chemicals and reagents

Unless otherwise noted, all chemicals and reagents were obtained from Sigma Aldrich (Saint Louis, MO, USA). Plasmid purification and gel extraction kits were obtained from iNtRON Biotechnology (Seoul, South Korea). Restriction enzymes and DNA ligase were purchased from Thermo Fisher Scientific (Austin, TX, USA). Graphene oxide (GO) nanosheets were synthesized from graphite in solution using a modified Hummers’ method^56–58^. Previous studies using similar modified Hummers’ methods resulted in 54–59% oxygenated carbons out of total carbon, according to XPS^12,57^. Hydrogen peroxide was added dropwise to reduce residual permanganate. The graphite oxide was thoroughly washed multiple times with pure DI water to eliminate residual strong organic acids and other Hummers’ reagents. The, graphite oxide was dried, resuspended in DI water, and exfoliated into GO nanosheets. Solutions of GO nanosheets were diluted to a concentration of 100 ppm, confirmed by dry weight.

### Synthesis and Purification of Mfp

The two proteins used in this study (Mfp5 and Mfp5^(3)^) were designed and recombinantly expressed using the methods from our previous study (Supplementary Table S1–S4)^27^. Mfp5 was directly expressed in *E. coli* strain BL21 (DE3). Mfp5^(3)^ was post-translationally spliced together in vitro from an Mfp5 protein with a C-terminal Cfa^N^ split intein domain and an Mfp5^(2)^ protein with an N-terminal Cfa^C^ split intein domain. Both Mfp5 and Mfp5^(3)^ proteins were purified using nickel affinity chromatography columns and were reacted with mushroom tyrosinase to convert tyrosine residues to DOPA residues. After purification and conversion of tyrosine residues, proteins were finally dialyzed in 0.5% v/v acetic acid.

### Synthesis of GO/Mfp film composites

Approximately 50 mL of GO solution (containing ~ 5 mg GO) was mixed with 1 mg of either Mfp5 or Mfp5^(3)^ protein suspended in 0.5% acetic acid (or equal volume of 0.5% acetic acid for a pure GO control film). If necessary, pH was adjusted to 4.5 with additional acetic acid. The mixture is sonicated on ice for a total of ~ 2 h cycling between 6 s on and 4 s off. After sonication, the solution was poured on top of a PES support membrane (Sterlitech, Kent, WA) inside an Advantech glass microanalysis filter holder (Cole-Palmer, Vernon Hills, IL). The solution was passed through the membrane using vacuum filtration. After filtration, the GO film was soft-baked at 37 °C for at least one hour, then peeled off the PES membrane.

### X-ray diffraction (XRD)

XRD patterns were obtained with a Rigaku Geigerflex X-ray powder diffractometer (Rigaku, Tokyo, Japan) with incident X-ray wavelength of λ = 1.506 Å, operating at 1.5 kV. The spectra were recorded from 5° to 50° (2θ) using a Cu Kα X-ray source.

### Thermogravimetric analysis (TGA)

A 100 μL platinum**-**high temperature pan (TA Instruments, New Castle, DE) was tared and GO film and purified lyophilized protein crosslinker samples were weighed prior to heating using a Q5000 IR thermogravimetric analyzer (TA Instruments). All measurements were conducted in nitrogen (AirGas, Radnor, PA) at a purge flow rate of 25 mL/min over a temperature range of 30–750 °C with a ramp rate of 10 °C/min.

### Scanning electron microscopy (SEM)

Films were mounted on a stainless steel sample holder using black carbon tape as an adhesive backing. Samples were coated with 10 nm Au using a Leica EM ACE600 high-vacuum sputter coater (Leica Microsystems, Wetzlar, Germany). The films were imaged with the Nova NanoSEM 230 field emission scanning electron microscope (Field Electron and Ion Company, FEI, Hillsboro, Oregon).

### Fourier transform infrared spectroscopy (FTIR)

FTIR spectra of the samples were collected using a Thermo Nicolet Nexus 470 (Thermo Scientific, Waltham, MA) following previous methods^59,60^. Specifically, spectra were acquired between wavenumbers of 500 cm^−1^ and 4000 cm^−1^. Peaks were assigned and compared to specific bonds according to previous studies of similar materials^12,24,55,61–63^.

### Mechanical testing

Mechanical properties, such as ultimate tensile strength and toughness, were measured using an MTS Criterion Model 41 universal test frame fitted with a 25 N load cell (MTS Systems Corporation, Eden Prairie, MN). Tests were conducted at a crosshead speed of 2.5 mm/min. The maximum force at fracture was divided by the cross-sectional area of the film strip to determine the ultimate tensile strength.

## Supplementary information

Supplementary Information.

## References

[1] Fiori G (2014). Electronics based on two-dimensional materials. Nat. Nanotechnol..

[2] Mahmoudi T, Wang Y, Hahn Y (2018). Graphene and its derivatives for solar cells application. Nano Energy.

[3] Neumaier D, Pindl S, Lemme MC (2019). Integrating graphene into semiconductor fabrication lines. Nat. Mater..

[4] Boretti A (2018). Outlook for graphene-based desalination membranes. npj Clean Water.

[5] Karim N (2017). Scalable production of graphene-based wearable E-textiles. ACS Nano.

[6] Kang S (2019). 2D reentrant micro-honeycomb structure of graphene-CNT in polyurethane: High stretchability, superior electrical/thermal conductivity, and improved shape memory properties. Compos. Part B.

[7] Wang J, Mu X, Sun M (2019). The thermal, electrical and thermoelectric properties of graphene nanomaterials. Nanomaterials.

[8] Zhang K, Zhang Y, Wang S (2013). Enhancing thermoelectric properties of organic composites through hierarchical nanostructures. Sci. Rep..

[9] Najafi F, Rajabi M (2015). Thermal gravity analysis for the study of stability of graphene oxide–glycine nanocomposites. Int. Nano Lett..

[10] Gao R (2013). Paper-like grapheme–Ag composite films with enhanced mechanical and electrical properties. Nanoscale Res. Lett..

[11] Zhu W (2017). High performances of artificial nacre-like graphene oxide carrageenan bio-nanocomposite films. Materials (Basel).

[12] Kim C, An S, Lee J, Zeng Q, Fortner JD (2018). Engineering graphene oxide laminate membranes for enhanced flux and boron treatment with polyethylenimine (PEI) polymers. ACS Appl. Mater. Interfaces.

[13] Park S, Dikin DA, Nguyen ST, Ruoff RS (2009). Graphene oxide sheets chemically cross-linked by polyallylamine. J. Phys. Chem. C.

[14] Liu X (2019). Thiol-branched graphene oxide and polydopamine-induced nanofibrillated cellulose to strengthen protein-based nanocomposite films. Cellulose.

[15] Crosby AJ, Lee J-Y (2007). Polymer nanocomposites: The “nano” effect on mechanical properties. Polym. Rev..

[16] Podsiadlo P (2007). Ultrastrong and stiff layered polymer nanocomposites. Science.

[17] Yoo SC, Lee J, Hong SH (2019). Synergistic outstanding strengthening behavior of graphene copper nanocomposites. Compos. Part B.

[18] Nuruddin M, Gupta R, Tcherbi-Narteh A, Hosur M, Jeelani S (2015). Synergistic effect of graphene nanoplatelets and nanoclay on epoxy polymer nanocomposites. Adv. Mater. Res..

[19] Tjong SC (2006). Structural and mechanical properties of polymer nanocomposites. Mater. Sci. Eng..

[20] Xie W (2018). Extreme mechanical behavior of nacre-mimetic graphene-oxide and silk nanocomposites. Nano Lett..

[21] Vural M (2017). Programmable molecular composites of tandem proteins with graphene oxide for efficient bimorph actuators. Carbon N. Y..

[22] Zhao G (2018). Reduced graphene oxide functionalized nano fibrous silk fibroin matrices for engineering excitable tissues. NPG Asia Mater..

[23] Jiang X, Li Z, Yao J, Shao Z, Chen X (2016). One-step synthesis of soy protein/graphene nanocomposites and their application in photothermal therapy. Mater. Sci. Eng. C.

[24] Xu X, Jiang X-Y, Jiao F-P, Chen X-Q, Yu J-G (2018). Tunable assembly of porous three-dimensional graphene oxide-corn zein composites with strong mechanical properties for adsorption of rare earth elements. J. Taiwan Inst. Chem. Eng..

[25] Mansoori E, Behzad T, Shafieizadegan-Esfahani AR (2019). Preparation and characterization of corn starch/soy protein biocomposite film reinforced with graphene and graphene oxide nanoplatelets. Polym. Adv. Technol..

[26] Alava T (2013). Control of the graphene−protein interface is required to preserve adsorbed protein function. Anal. Chem..

[27] Kim E (2018). Microbially synthesized repeats of mussel foot protein display enhanced underwater adhesion. ACS Appl. Mater. Interfaces.

[28] Anderson TH (2010). The contribution of DOPA to substrate–peptide adhesion and internal cohesion of mussel-inspired synthetic peptide films. Adv. Funct. Mater..

[29] Kim S (2015). Cation–π interaction in DOPA-deficient mussel adhesive protein mfp-1. J. Mater. Chem. B.

[30] Hofman AH, Van Hees IA, Yang J, Kamperman M (2018). Bioinspired underwater adhesives by using the supramolecular toolbox. Adv. Mater..

[31] Gebbie MA (2017). Tuning underwater adhesion with cation–π interactions. Nat. Chem..

[32] Akdogan Y (2014). Intrinsic surface-drying properties of bioadhesive proteins. Angew. Chem. Int. Ed..

[33] Wei W (2016). An underwater surface-drying peptide inspired by a mussel adhesive protein. Adv. Funct. Mater..

[34] Bowen CH (2018). Recombinant spidroins fully replicate primary mechanical properties of natural spider silk. Biomacromol.

[35] Jenkins CL, Meredith HJ, Wilker JJ (2013). Molecular weight effects upon the adhesive bonding of a mussel mimetic polymer. ACS Appl. Mater. Interfaces.

[36] Hwang DS, Yoo HJ, Jun JH, Moon WK, Cha HJ (2004). Expression of functional recombinant mussel adhesive protein Mgfp-5 in *Escherichia coli*. Appl. Environ. Microbiol..

[37] Li Y-Q, Yu T, Yang T-Y, Zheng L-X, Liao K (2012). Bio-inspired nacre-like composite films based on graphene with superior mechanical, electrical, and biocompatible properties. Adv. Mater..

[38] Putz KW, Compton OC, Palmeri MJ, Nguyen ST, Brinson LC (2010). High-nanofiller-content graphene oxide–polymer nanocomposites via vacuum-assisted self assembly. Adv. Funct. Mater..

[39] Dikin DA (2007). Preparation and characterization of graphene oxide paper. Nature.

[40] Üst UC, Demir SB, Dağci K, Alanyalioğlu M (2016). Fabrication of free-standing graphene paper decorated with flower-like PbSe_0.5_S_0.5_ structures. RSC Adv..

[41] Zaid NAM, Idris NH (2016). Enhanced capacitance of hybrid layered graphene/nickel nanocomposite for supercapacitors. Sci. Rep..

[42] Gascho JLS, Costa SF, Recco AAC, Pezzin SH (2019). Graphene oxide films obtained by vacuum filtration: X-ray diffraction evidence of crystalline reorganization. J. Nanomater..

[43] Talyzin AV, Hausmaninger T, You S, Szabó T (2014). The structure of graphene oxide membranes in liquid water, ethanol and water–ethanol mixtures. Nanoscale.

[44] Lerf A (2006). Hydration behavior and dynamics of water molecules in graphite oxide. J. Phys. Chem. Solids.

[45] Li W, Wu W, Li Z (2018). Controlling interlayer spacing of graphene oxide membranes by external pressure regulation. ACS Nano.

[46] Rezania B, Severin N, Talyzin AV, Rabe JP (2014). Hydration of bilayered graphene oxide. Nano Lett..

[47] Kang SM (2011). Simultaneous reduction and surface functionalization of graphene oxide by mussel-inspired chemistry. Adv. Funct. Mater..

[48] Ashby M (2016). Materials Selection in Mechanical Design.

[49] Park S (2008). Graphene oxide papers modified by divalent ions-enhancing mechanical properties via chemical cross-linking. ACS Nano.

[50] Tian Y, Cao Y, Wang Y, Yang W, Feng J (2013). Realizing ultrahigh modulus and high strength of macroscopic graphene oxide papers through crosslinking of mussel-inspired polymers. Adv. Mater..

[51] Perreault F, Fonseca De Faria A, Elimelech M (2015). Environmental applications of graphene-based nanomaterials. Chem. Soc. Rev..

[52] Li F, Jiang X, Zhao J, Zhang S (2015). Graphene oxide: A promising nanomaterial for energy and environmental applications. Nano Energy.

[53] Jiang Y, Biswas P, Fortner JD (2016). A review of recent developments in graphene-enabled membranes for water treatment. Environ. Sci. Water Res. Technol..

[54] Han L (2018). Mussel-inspired adhesive and conductive hydrogel with long-lasting moisture and extreme temperature tolerance. Adv. Funct. Mater..

[55] Cui W (2014). A strong integrated strength and toughness artificial nacre based on dopamine cross-linked graphene oxide. ACS Nano.

[56] Hummers WS, Offeman RE (1958). Preparation of graphitic oxide. J. Am. Chem. Soc..

[57] Jiang Y, Zeng Q, Biswas P, Fortner JD (2019). Graphene oxides as nanofillers in polysulfone ultrafiltration membranes: Shape matters. J. Membr. Sci..

[58] Jiang Y (2015). Engineered crumpled graphene oxide nanocomposite membrane assemblies for advanced water treatment processes. Environ. Sci. Technol..

[59] Dai B, Sargent CJ, Gui X, Liu C, Zhang F (2019). Fibril self-assembly of amyloid-spider silk block polypeptides. Biomacromol.

[60] Bowen CH (2019). Seeded chain-growth polymerization of proteins in living bacterial cells. ACS Synth. Biol..

[61] Duan J (2016). Bioinspired ternary artificial nacre nanocomposites based on reduced graphene oxide and nanofibrillar cellulose. ACS Appl. Mater. Interfaces.

[62] Jiang Q (2016). Bilayered biofoam for highly efficient solar steam generation. Adv. Mater..

[63] Eigler S, Dotzer C, Hirsch A, Enzelberger M, Müller P (2012). Formation and decomposition of CO_2_ intercalated graphene oxide. Chem. Mater..

