# Supplementary material for: Graphene oxide/mussel foot protein composites for high-strength and ultra-tough thin films
Source: Sci Rep. 2020 Nov 5;10:19082. doi: 10.1038/s41598-020-76004-6 (PMC7644685; doi:10.1038/s41598-020-76004-6)
Supplement: Supplementary file 1 — Supplementary Information. [file 41598_2020_76004_MOESM1_ESM.docx]

**SUPPLEMENTARY INFORMATION**

**Graphene Oxide/Mussel Foot Protein Composites for High-Strength and Ultra-Tough Thin Films**

*Eugene Kim ^1^, Xuyan Qin ^1^, James B. Qiao ^1^, Qingqing Zeng ^1^, John D. Fortner ^2^, Fuzhong Zhang ^1,3,4,*^*

^1^Department of Energy, Environmental & Chemical Engineering, Washington University in St. Louis, Saint Louis, MO 63130

^2^Department of Chemical & Environmental Engineering, Yale University, New Haven, CT 06520

^3^Institute of Materials Science and Engineering, Washington University in St. Louis, Saint Louis, MO 63130

^4^Division of Biological & Biomedical Sciences, Washington University in St. Louis, Saint Louis, MO 63130

One Brookings Drive, Saint Louis, Missouri 63130; telephone: (314) 935-7671; fax: (314) 935-7211;

*Corresponding author: fzhang@seas.wustl.edu

**Table S1. Coding sequences used in this study**

| **Name** | **Sequence** | **Description** | **Source** |
| --- | --- | --- | --- |
| **mfp5** | GCTAAGACTAAA**CATCATCACCATCACCAC**GGTGGCGGTGGCAGCAGATCTGgatctAGCAGCGAAGAGTATAAAGGTGGTTACTACCCGGGCAACACTTACCACTACCATAGCGGCGGCTCCTACCACGGTTCCGGCTATCACGGTGGCTACAAAGGTAAATACTACGGCAAAGCGAAAAAGTACTACTACAAATATAAAAACTCCGGCAAATACAAGTATCTGAAGAAGGCTCGTAAATATCACCGTAAAGGTTACAAAAAGTATTACGGCGGTGGTTCTTCTgGATCC | Coding sequence encoding Mfp5 protein, including coding sequences for AKTK expression tag and **His_6_ affinity tag** | Kim et al. 2018^1^ |
| **A-mfp5^(2)^-cfa^N^** | GCTAAGACTAAAGGTACCTCAAGTGAAGAATATAAGGGCGGCTATTACCCTGGGAACACCTACCATTACCACTCGGGTGGTTCGTATCATGGTTCCGGCTACCATGGTGGTTACAAGGGGAAGTATTATGGTAAAGCGAAGAAATACTATTACAAATACAAGAATTCTGGAAAGTATAAATATCTGAAAAAAGCTCGCAAATATCATCGTAAAGGATATAAAAAATACTATGGAGGTGGCAGTAGCAGTTCCGAAGAATATAAAGGGGGTTACTACCCTGGAAACACTTATCATTATCATAGTGGAGGTTCTTACCATGGCAGTGGATATCACGGCGGTTATAAGGGTAAGTATTATGGGAAAGCCAAAAAGTATTACTATAAGTACAAGAATTCTGGCAAATACAAGTACTTGAAGAAGGCTCGTAAGTACCACCGCAAAGGTTACAAGAAATACTATGGAGGCGGTTCAAGTTCCGGA**GCAGAATATTGCCTGTCTTACGACACAGAGATTCTGACCGTTGAATATGGATTCCTTCCTATCGGTAAGATCGTGGAGGAACGGATTGAATGCACAGTCTATACGGTAGATAAAAATGGCTTTGTGTATACACAACCTATTGCTCAGTGGCATAACCGGGGAGAACAGGAAGTTTTCGAATACTGCTTAGAAGACGGTTCGATTATCCGTGCAACGAAAGATCACAAATTTATGACGACCGACGGTCAGATGTTACCGATTGATGAGATTTTCGAACGGGGGTTAGACCTGAAACAAGTTGATGGTTTGCCG** | Coding sequence encoding Mfp5^(2)^ protein, including AKTK expression tag and **Cfa^N^ intein & native extein** | Kim et al. 2018^1^ |
| **cfa^C^-mfp5-H_10_** | **GTCAAGATCATTAGTCGTAAGAGTCTGGGCACTCAAAACGTCTACGATATTGGAGTAGAAAAAGATCATAATTTTTTGCTGAAGAATGGGCTGGTGGCCTCTAACTGCTTCAAC**GGTACCAGCAGCGAAGAGTATAAAGGTGGTTACTACCCGGGCAACACTTACCACTACCATAGCGGCGGCTCCTACCACGGTTCCGGCTATCACGGTGGCTACAAAGGTAAATACTACGGCAAAGCGAAAAAGTACTACTACAAATATAAAAACTCCGGCAAATACAAGTATCTGAAGAAGGCTCGTAAATATCACCGTAAAGGTTACAAAAAGTATTACGGCGGTGGTTCTTCTTCCGGAGGTGGACATCACCATCACCATCATCACCACCATCAC | Coding sequence encoding Mfp5 protein, including **Cfa^C^ intein & native extein**, and H_10_ affinity tag | Kim et al. 2018^1^ |

**Table S2. Plasmids used in this study**

| **Name** | **ORI** | **Promoter** | **Resistance** | **Gene** | **Source** |
| --- | --- | --- | --- | --- | --- |
| **pE7a-mfp5** | ColE1 | P_T7_ | Amp^R^ | mfp5 | Kim et al. 2018^1^ |
| **pE7a-mfp5^(2)^-Cfa^N^** | ColE1 | P_T7_ | Amp^R^ | mfp5^(2)^ + Cfa^N^ | Kim et al. 2018^1^ |
| **pE7a-Cfa^C^-mfp5** | ColE1 | P_T7_ | Amp^R^ | Cfa^C^ + mfp5 | Kim et al. 2018^1^ |

**Table S3. Strains used in this study**

| **Name** | **Genotype** | **Source** |
| --- | --- | --- |
| **sB-Mfp5** | BL21(DE3) containing pE7a-mfp5 | Kim et al. 2018^1^ |
| **sB-Mfp5^(2)^-Cfa^N^** | BL21(DE3) containing pE7a-mfp5^(2)^-Cfa^N^ | Kim et al. 2018^1^ |
| **sB-Cfa^C^-Mfp5** | BL21(DE3) containing pE7a-Cfa^C^-mfp5 | Kim et al. 2018^1^ |

**Table S4. Protein sequences used in this study**

| **Name** | **Sequence** | **Description** | **Source** |
| --- | --- | --- | --- |
| **Mfp5** | AKTK**HHHHHH**GGGGSRSGSSSEEYKGGYYPGNTYHYHSGGSYHGSGYHGGYKGKYYGKAKKYYYKYKNSGKYKYLKKARKYHRKGYKKYYGGGSSGS | Mfp5 protein, including AKTK expression tag and **His_6_ affinity tag** | Kim et al. 2018^1^; Hwang et al, 2004^2^ |
| **A-Mfp5^(2)^-Cfa^N^** | AKTKGTSSEEYKGGYYPGNTYHYHSGGSYHGSGYHGGYKGKYYGKAKKYYYKYKNSGKYKYLKKARKYHRKGYKKYYGGGSSSSEEYKGGYYPGNTYHYHSGGSYHGSGYHGGYKGKYYGKAKKYYYKYKNSGKYKYLKKARKYHRKGYKKYYGGGSSSG**AEYCLSYDTEILTVEYGFLPIGKIVEERIECTVYTVDKNGFVYTQPIAQWHNRGEQEVFEYCLEDGSIIRATKDHKFMTTDGQMLPIDEIFERGLDLKQVDGLP** | Mfp5^(2)^ protein, including AKTK expression tag and **Cfa^N^ intein & native extein** | Kim et al. 2018^1^; Stevens et al. 2016^3^ |
| **Cfa^C^-Mfp5-H_10_** | **VKIISRKSLGTQNVYDIGVEKDHNFLLKNGLVASNCFN**GTSSEEYKGGYYPGNTYHYHSGGSYHGSGYHGGYKGKYYGKAKKYYYKYKNSGKYKYLKKARKYHRKGYKKYYGGGSSSGGGHHHHHHHHHH | Mfp5 protein, including **Cfa^C^ intein & native extein**, and H_10_ affinity tag | Kim et al. 2018^1^; Stevens et al. 2016^3^ |


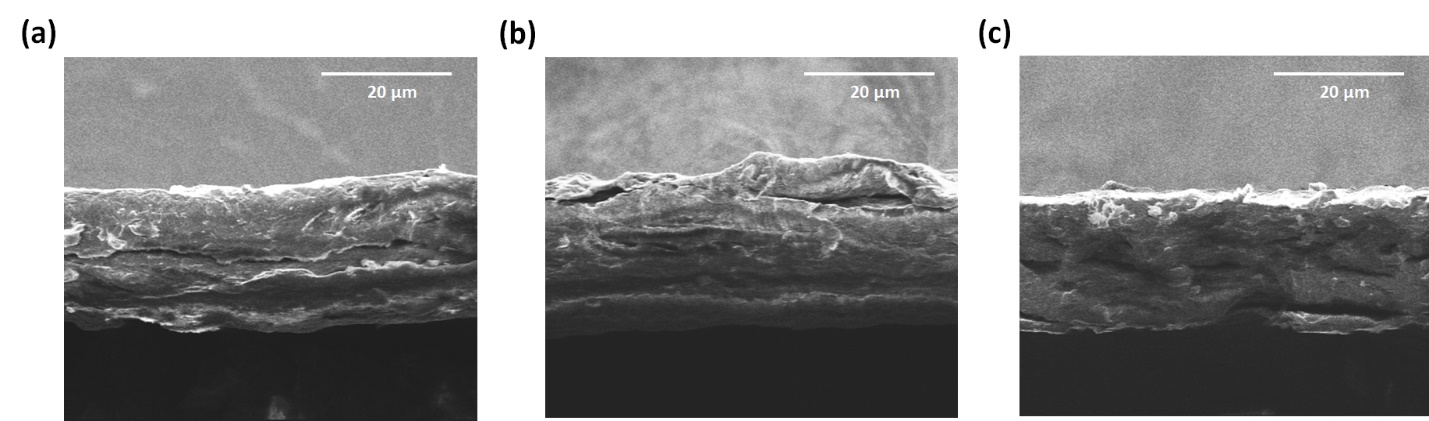


**Figure S1. SEM images.** GO-Mfp5 after (a) 3 days, (b) 4 days, and (c) 5 days of vacuum filtration.


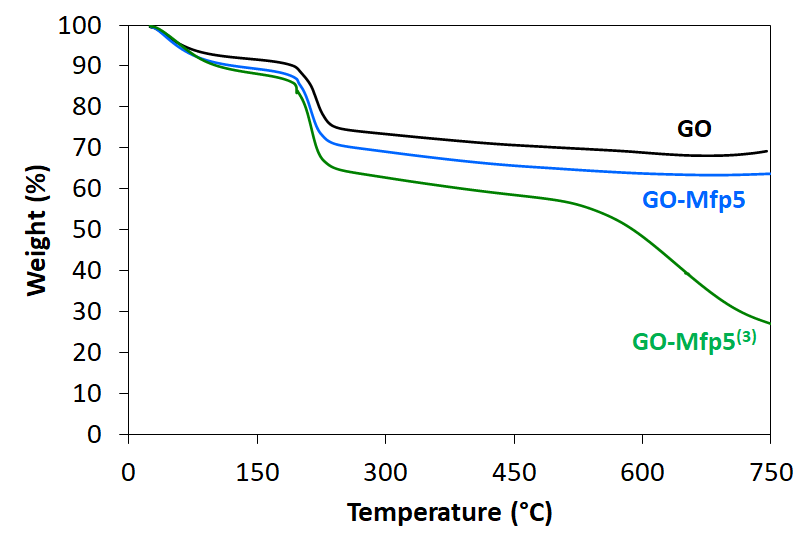


**Figure S2. TGA curves of pure GO and composite films.** All films are extremely stable up to 550 °C, which is significantly higher than the melting point of pure proteins. Approximately 2-5 mg of sample was used for each experiment.


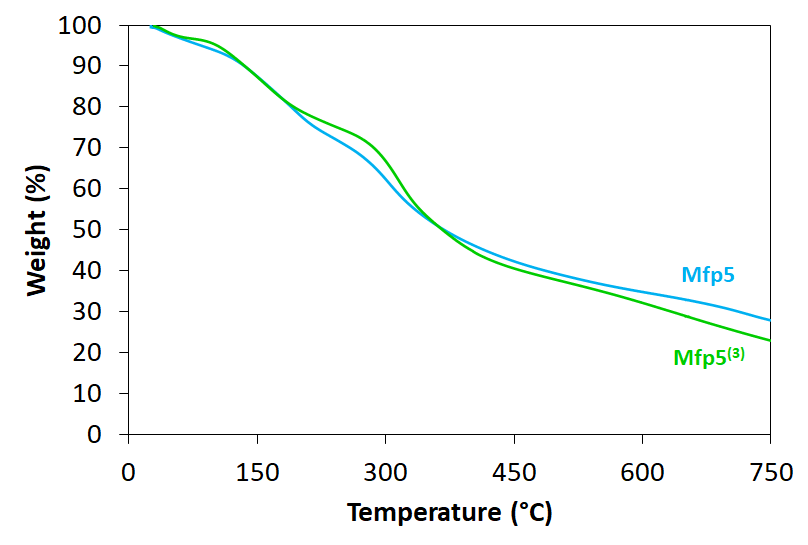


**Figure S3. TGA curves of pure Mfp5 and Mfp5^(3)^ proteins.** Approximately 2-5 mg of each protein was used for each experiment.


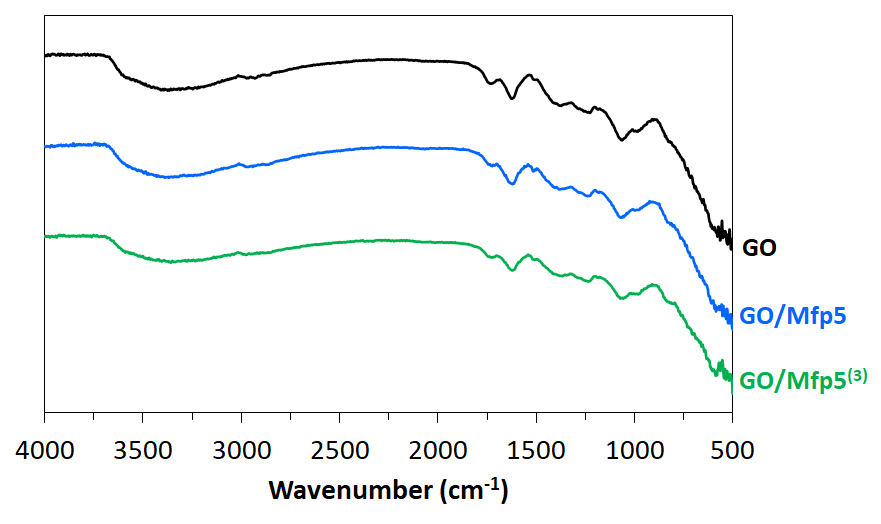


**Figure S4. FTIR spectra of pure GO, GO/Mfp5 and GO/Mfp5^(3)^ composite films.** All spectra match characteristic peaks of graphene oxide and show no peak shifts or peak formation relative to each other, suggesting that there are no new covalent interactions between the film components.

**REFERENCES**

1. Kim, E. *et al.* Microbially Synthesized Repeats of Mussel Foot Protein Display Enhanced Underwater Adhesion. *ACS Appl. Mater. Interfaces* **10**, 43003–43012 (2018).

2. Hwang, D. S., Yoo, H. J., Jun, J. H., Moon, W. K. & Cha, H. J. Expression of functional recombinant mussel adhesive protein Mgfp-5 in Escherichia coli. *Appl. Environ. Microbiol.* (2004). doi:10.1128/AEM.70.6.3352-3359.2004

3. Stevens, A. J. *et al.* Design of a Split Intein with Exceptional Protein Splicing Activity. *J. Am. Chem. Soc.* **138**, 2162–2165 (2016).
